# The annotation of genomic dataset sequences of the sugar beet root maggot *Tetanops myopaeformis*, TmSBRM_v1.0

**DOI:** 10.1016/j.dib.2024.110710

**Published:** 2024-07-09

**Authors:** Sudha Acharya, Nadim W. Alkharouf, Chenggen Chu, Vincent P. Klink

**Affiliations:** a Department of Computer and Information Sciences, Towson University, Towson, MD, 21252, USA; b USDA-ARS-NA- Northern Great Plains Research Laboratory, 1307N 18TH ST, Northern Crop Science Laboratory, Fargo, ND 58102, USA; c USDA-ARS-NEA-BARC, Molecular Plant Pathology Laboratory, Building 004, Room 122, BARC-West, 10300 Baltimore Ave., Beltsville, MD 20705, USA

**Keywords:** Genome, Plant pathogen, Insect, Evolution, *Drosophila*, Climate change, Agronomic, Stakeholder

## Abstract

*Tetanops myopaeformis*, the sugar beet root maggot (SBRM), is a devastating insect pathogen of sugar beet, one of only two plants in the world from which sugar is widely produced, accounting for 55% of U.S. sugar and 35% of global raw sugar with an annual farm value of $3 billion in the United States. *T. myopaeformis* is capable of causing total crop failure, making its study important. The previously released SBRM genome, TmSBRM_v1.0, has been generated from the *de novo* assembled draft genome sequence of *T. myopaeformis* isolated that was isolated from field-grown *B. vulgaris* in North Dakota, USA. The annotation of the *T. myopaeformis* is presented here. The annotated *T. myopaeformis* genome should be useful in understanding the biology of this insect and the development of new control strategies for this pathogen, relationship to model genetic organisms like *Drosophila melanogaster* and aid in agronomic improvement of sugar beet for stakeholders while also providing information on the relationship between the SBRM and climate change.

Specifications Table

**Table:** 

Subject	Omics
Specific subject area	Genomics
Data format	Raw, Analyzed, Filtered
Type of data	Table, Figures
Data collection	
Data source location	Fargo, ND, USA
Data accessibility	Repository name: NCBI Data identification number: BioSample accession: SAMN37733483, BioProject ID PRJNA1026092. Direct URL to data: https://www.ncbi.nlm.nih.gov/bioproject/PRJNA1026092 The data has been deposited in Genbank SRA archive found at NCBI, meeting their requirements for submission.

## 1 Value of the Data

• The annotation of the *T. myopaeformis*, SBRM genome, TmSBRM_v1.0, will provide data that researchers can use to understand the fly's biology. The information will allow for gaining knowledge on chromosome structure, genome organization, maintenance, and regulation. The information will allow for an understanding of gene expression which will likely include sex specific gene expression regulation. Furthermore, the annotation will be useful for understanding insect evolution, as well as the development and evolution of a pathological niche. The information will aid in the understanding of host selection, ecology, climate change. The SBRM genome annotation will aid in other aspects of improving the understanding of their biology, and agronomic impact(s). The information and methods will aid in future genome sequencing efforts, and annotations.
• The data which is deposited in a public database is available freely for use.
• The anticipated use of the data is scientific in nature. The annotation will allow for an understanding of genes, and gene family composition. The annotation will permit the identification of targets for the suppression of essential pathogen gene function through overexpression, RNA interference (RNAi), mutagenesis, or gene editing through clustered regularly interspaced short palindromic repeats (CRISPR)/CRISPR-associated protein 9 (Cas9) (CRISPR/Cas9)-mediated processes that synthetically modify genes [[1], [2], [3], [4], [5]]; Jinek et al. 2012; [[6], [7], [8], [9]]. The annotation of the TmSBRM_v1.0 reference genome allows for a better scientific investigation of the insect, and related insect species that have pathogenic life cycles.

## 2 Background

*Tetanops myopaeformis* (von Röder), the sugar beet root maggot (SBRM), is a devastating pathogen of sugar beet (SB), *Beta vulgaris*, ssp vulgaris (*B. vulgaris*) [10,11]. SB is an important food crop, while also being one of only two plants globally from which sugar is widely produced, and accounting for 35% of global raw sugar with an annual farm value of $3 billion in the United States alone [10,11]. SBRM is the most devastating pathogen of sugar beet in North America [1,12]. Agricultural control of SBRM is limited by a scarcity of genetic knowledge. Recently, a *de novo* sequenced and assembled draft genome of *T. myopaeformis* was presented [12]. The analysis presented here is aimed at providing an annotation of that genome resource for the scientific community and a basis for future updates. The work describes the data, by providing genome statistics, including the gene's biological process, cellular component, and molecular function. A list of mobile elements is provided (McClintock, 1951). The work explains its utility to the community, provides protocols and references, and provides a documented link to the data that is in a standard, re-useable format. The annotation identified numerous genes associated with other pathogenic organisms, including insects, which may provide useful information in understanding the pathogenic nature of *T. myopaeformis* that could be used to devise strategies to control this devastating pathogen.

## 3 Data Description

**Fig. 1:**
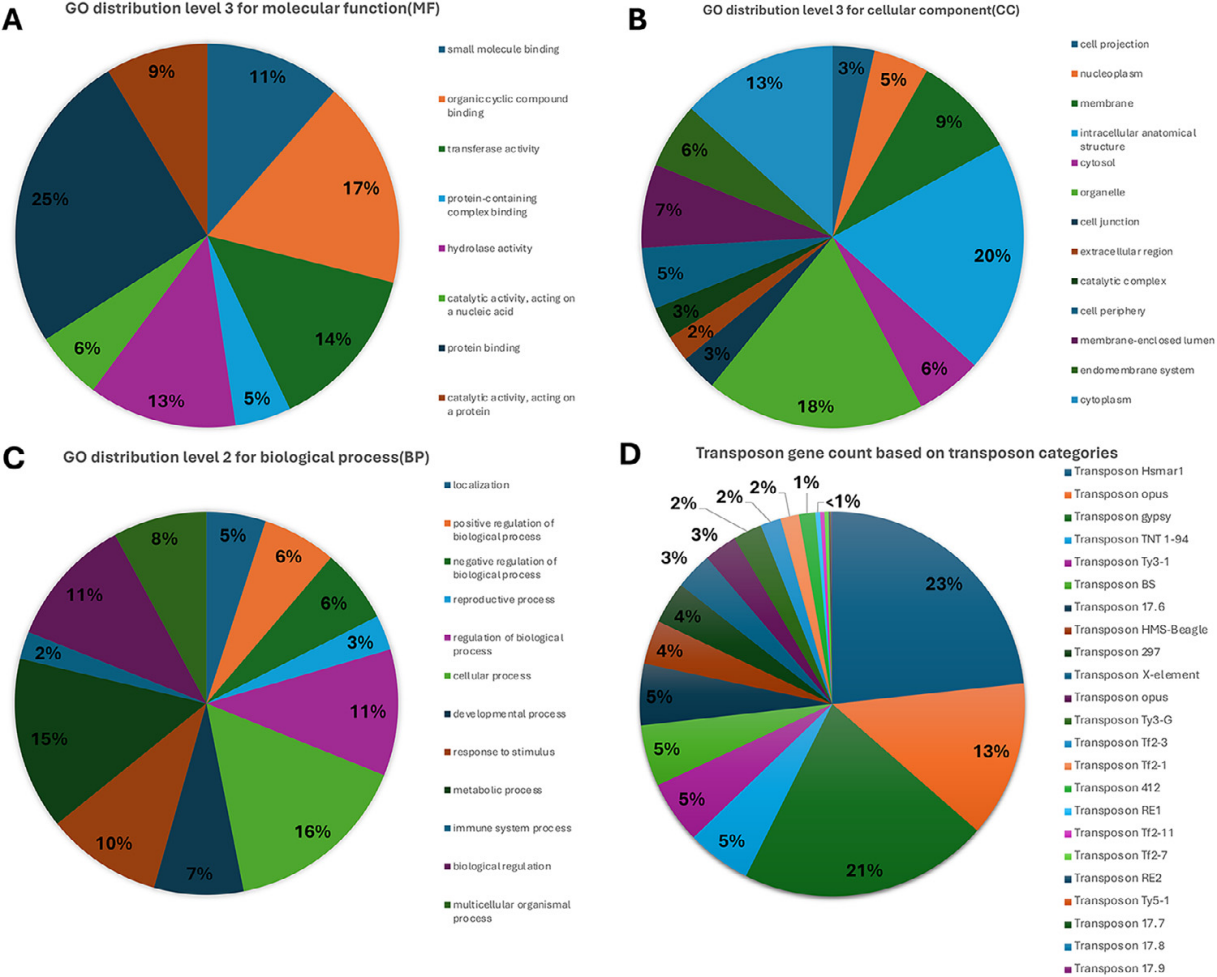
Gene annotation. A. Biological process. B. Cellular component. C. Molecular function. D. Transposable elements.

An annotation of the *de novo* assembly of the *T. myopaeformis* genome is presented for BioSample accession: SAMN37733483, BioProject ID PRJNA1026092. The data are at the URL: https://www.ncbi.nlm.nih.gov/bioproject/PRJNA1026092. Statistics for the annotation are provided (Table 1). This information may aid in understanding basic aspects of the genome's composition. The blast matches to a particular organism are provided (Supplemental Table 2). This information may provide useful information relating to the pathogenic life stages that may not be obtained from an annotation limited to comparisons to *D. melanogaster* that is not parasitic. We provide annotations for biological process (Fig. 1). This information may provide useful information regarding the protein product's role. We provide annotations for cellular components (Fig. 1). This information may provide useful information regarding cellular or subcellular involvement. We provide annotations for molecular function (Fig. 1). This information may provide specific information on the role of the protein product. A large number of transposons were identified (Fig. 1). This information may be useful in understanding genome regulation, evolution, and plasticity. The files related to the annotations are provided as supplementary information (Supplemental Table 1). This information is provided so the basis of the analyses is available.

**Table 1:** Genome statistics.

Statistics of gene information	
% GC content	42.20%
Min sequence length	193
Max sequence length	110,084
average length	612.8
Total number of sequences	28,276

## 4 Experimental Design, Materials and Methods

The *T. myopaeformis* genome (https://www.ncbi.nlm.nih.gov/bioproject/PRJNA1026092) was *de novo* sequenced and archived at Genbank (Benson et al. 2012; [12]). The genome sequence was obtained from NCBI [12]. Alkharouf et al. [12] used the pipeline Flye, version 2.9.2, to assemble the PacBio HiFi DNA reads using default values, except for setting the –asm-coverage argument to 50, to reduce memory consumption. The gene finding tool AUGUSTUS 3.5.0 (Hoff et al.2019) was used for gene prediction analysis in these contigs with the complete gene option enabled and default set for the rest of the parameters. *Drosophila melanogaster* is the most closely related genetic model that was most closely related, phylogenetically, to *T. myopaeformis* so it was used as the reference species to *T. myopaeformis* TmSBRM_v1.0. The identified genes were used to perform a blast against the non-reductant (NR) database to predict genes. For generating the gene functional annotation, the predicted genes were functionally annotated using Blast2GO 6.0 using default values [13]. The gene model was blasted as blastp against the NCBI NR protein database. InterproScan 5.67–99.0 (Zdobnov et al. 2001) was used under default values for domain finding. Then GO mapping and annotation was performed under default values using GeneOntology 2024–03–28 [14].

## Limitations

The annotation represents DNA isolated from 5 individuals. All the genetic variation that occurs within and between populations has likely not been captured.

## Ethics Statement

The authors have read and follow the ethical requirements for publication in Data in Brief and confirming that the current work does not involve human subjects, animal experiments, or any data collected from social media platforms.

## CRediT authorship contribution statement

**Sudha Acharya:** Methodology, Software, Validation, Formal analysis, Investigation, Resources, Data curation, Writing – original draft. **Nadim W. Alkharouf:** Methodology, Software, Validation, Formal analysis, Investigation, Resources, Data curation, Writing – original draft. **Chenggen Chu:** Investigation, Resources. **Vincent P. Klink:** Conceptualization, Methodology, Resources, Visualization, Supervision, Project administration, Funding acquisition, Writing – original draft.

## Data Availability

TmSBRM_v1.0 (Reference data) (NCBI).

## Appendix Supplementary materials

Supplemental data 1. Information relating to genome statistics, blast information, functional annotation, blast hit genes, characterized genes, and transposons. Image, application 1
